# Case-Control Study Examining the Association between Selective Serotonin Reuptake Inhibitors Use and Hepatocellular Carcinoma

**DOI:** 10.3389/fphar.2017.00861

**Published:** 2017-11-22

**Authors:** Shih-Wei Lai, Kuan-Fu Liao, Cheng-Li Lin, Hsien-Feng Lin

**Affiliations:** ^1^Department of Medicine, China Medical University, Taichung, Taiwan; ^2^Department of Family Medicine, China Medical University Hospital, Taichung, Taiwan; ^3^Department of Medicine, Tzu Chi University, Hualien, Taiwan; ^4^Department of Internal Medicine, Taichung Tzu Chi General Hospital, Taichung, Taiwan; ^5^Management Office for Health Data, China Medical University Hospital, Taichung, Taiwan; ^6^Department of Chinese Medicine, China Medical University, Taichung, Taiwan

**Keywords:** hepatocellular carcinoma, selective serotonin reuptake inhibitors, Taiwan, National Health Insurance Program, case-control study

## Abstract

**Objectives:** The purpose of the study was to assess the relationship between selective serotonin reuptake inhibitors use and hepatocellular carcinoma in Taiwan.

**Methods:** Using the database of the Taiwan National Health Insurance Program, we conducted a case-control study to identify 4901 subjects aged 20 years and more with newly diagnosed hepatocellular carcinoma in 2000–2013 as the cases. We randomly selected 19604 subjects aged 20 years and more without hepatocellular carcinoma as the controls. Both cases and controls were matched with sex and age. Ever use of selective serotonin reuptake inhibitors was defined as a subject who had at least a prescription for selective serotonin reuptake inhibitors before index date. Never use was defined as a subject who never had a prescription for selective serotonin reuptake inhibitors before index date. The odds ratio (OR) and 95% confidence interval (CI) for hepatocellular carcinoma associated with selective serotonin reuptake inhibitors use was estimated by the multivariable logistic regression model.

**Results:** Among subjects with any one of the comorbid conditions associated with hepatocellular carcinoma, the adjusted OR of hepatocellular carcinoma was 0.89 (95% CI 0.75, 1.06) for subjects with ever use of selective serotonin reuptake inhibitors, comparing with never use.

**Conclusion:** The findings indicate that among subjects with any one of the comorbid conditions associated with hepatocellular carcinoma, no significant association can be detected between selective serotonin reuptake inhibitors use and hepatocellular carcinoma.

## Introduction

Selective serotonin reuptake inhibitors are used to treat major depressive disorder and anxiety disorder (Hirschfeld, 2000; Baldwin et al., 2002; Hedges et al., 2007). Recently, an *in vitro* study showed that selective serotonin reuptake inhibitors seemed to have anti-tumor effects on human hepatocellular carcinoma (Kuwahara et al., 2015). A animal study showed that fluoxetine, one of selective serotonin reuptake inhibitors, was not associated with increased incidence of hepatocellular carcinoma in rats and mice (Bendele et al., 1992). Clinically, a cohort study showed that selective serotonin reuptake inhibitors use was not significantly associated with increased incidence of hepatocellular carcinoma (Haukka et al., 2010). Furthermore, a case-control study showed that selective serotonin reuptake inhibitors use was significantly associated with decreased odds for hepatocellular carcinoma (Chen et al., 2017). Inconsistent results exit on the relationship between selective serotonin reuptake inhibitors use and hepatocellular carcinoma.

Hepatocellular carcinoma was the second leading cause of cancer death in Taiwan in 2016 (Ministry of Health and Welfare, 2016a). In Taiwan, hepatitis B, hepatitis C, heavy alcohol consumption, and diabetes mellitus are associated with increased risk of hepatocellular carcinoma (Wang et al., 2003; Chen, 2007; Lai et al., 2012). Without these comorbid conditions, the likelihood of developing hepatocellular carcinoma is low in Taiwan. Therefore, any study exploring the drug effect on chemoprevention of hepatocellular carcinoma in Taiwan should make an adjustment for these comorbid conditions. To clarify this issue, we designed a population-based case-control study to explore whether there is a relationship between selective serotonin reuptake inhibitors use and hepatocellular carcinoma among persons with any one of these comorbid conditions associated with hepatocellular carcinoma.

## Materials and Methods

### Data Source

Taiwan is an independent country with more than 23 million citizens (Chan et al., 2016; Chang and Yu, 2016; Chang et al., 2016; Chen and Wu, 2016; Chen S.Y. et al., 2016; Chen Y.F. et al., 2016; Cheng et al., 2016a,b; Hsieh et al., 2016; Hsu and Yin, 2016; Huang and Chang, 2016; Lin and Lin, 2016; Maa and Leu, 2016; Ooi, 2016; Yu et al., 2016; Liang et al., 2017; Liao et al., 2017c; Wen and Yin, 2017). We conducted a population-based case-control study using the database of citizens enrolled in the Taiwan National Health Insurance Program. This insurance program began in March 1995 and the enrollment rate has exceeded 99.6% of the entire population of 23 million citizens living in Taiwan in 2015 (Ministry of Health and Welfare, 2016b). The study was approved by the Research Ethics Committee of China Medical University and Hospital in Taiwan (CMUH-104-REC2-115). The details of the program have been written down in previous studies (Lai et al., 2010; Cheng et al., 2012; Liao et al., 2012; Chen et al., 2013; Hung et al., 2013).

### Selection of Subjects

We identified subjects aged 20 years and more with newly diagnosed hepatocellular carcinoma (International Classification of Diseases, Ninth Revision, Clinical Modification, ICD-9 codes 155, 155.0, and 155.2) from 2000 to 2013 as the cases. The date of subjects being diagnosed with hepatocellular carcinoma was defined as the index date. Subjects without the diagnosis of hepatocellular carcinoma were randomly selected as the controls. Both cases and controls were matched with sex, age (5-year interval), and the year of index date. Subjects who had any other cancer (ICD-9 codes 140-208) before the index date were excluded from the study. The definition of subject selection was adapted from previous studies (Lai et al., 2013a,b).

### Comorbidities Related to Hepatocellular Carcinoma

Comorbidities which could be related to hepatocellular carcinoma were selected as follows: alcohol-related disease, cardiovascular disease, chronic kidney disease, chronic obstructive pulmonary disease, diabetes mellitus, hyperlipidemia, hypertension, as well as chronic liver disease including cirrhosis, hepatitis B, hepatitis C, and other chronic hepatitis. All comorbidities were diagnosed based on ICD-9 codes. The validity of ICD-9 codes has been discussed in previous studies (Hung et al., 2016; Lai et al., 2016, 2017a,b; Liao et al., 2016a,b, 2017a,b; Shen et al., 2016; Wong et al., 2016; Lin C.M. et al., 2017; Hung et al., 2017).

### Measurements of Selective Serotonin Reuptake Inhibitors Use and Other Medications Use

Prescription histories of selective serotonin reuptake inhibitors and other medications including metformin and statin were collected in the study. Selective serotonin reuptake inhibitors available in Taiwan during 2000–2013 were included as follows: fluoxetine, fluvoxamine, paroxetine, sertraline, citalopram, and escitalopram. The definition of medication use was found in previous studies (Cheng et al., 2017; Lai et al., 2017d,e; Liao et al., 2017d,e). That is, ever use of medications was classified as a subject who had at least a prescription of medications studied before the index date. Never use of medications was classified as a subject who never had a prescription of medications studied before the index date.

### Statistical Analysis

We assessed the differences of the demographic status, selective serotonin reuptake inhibitors use, other medications use, and comorbidities between the cases and the controls by using the Chi-square test for categorized variables. The *t*-test was used to examine the difference of mean age between the cases and the controls. Initially, all variables were included in the univariable logistic regression model. Only those significantly associated with hepatocellular carcinoma in the univariable logistic regression model could be further included in the multivariable logistic regression model. We measured the odds ratio (OR) and the 95% confidence interval (CI) for hepatocellular carcinoma associated with selective serotonin reuptake inhibitors use. All analyses were performed using the SAS statistical software (version 9.2; SAS Institute, Inc., Cary, NC, United States). The results were considered statistically significant when two-tailed *P*-values were less than 0.05.

## Results

### Characteristics of the Study Population

In **Table 1**, we identified 4901 cases with newly diagnosed hepatocellular carcinoma in 2000–2013 and 19604 controls. Both cases and controls had similar distributions of sex and age. The mean ages (standard deviation) were 62.5 (12.3) years in cases with hepatocellular carcinoma and 62.3 (12.4) years in controls, without statistical significance (*t*-test, *P* = 0.44). The cases with hepatocellular carcinoma were more likely to have a higher proportion of ever use of selective serotonin reuptake inhibitors than the controls (7.57% vs. 6.05%, *P* = 0.001). In addition, the proportions of ever use of metformin, alcohol-related disease, chronic kidney disease, chronic liver disease, chronic obstructive pulmonary disease, diabetes mellitus, and hypertension were higher in the cases than the controls, with statistical significance (*P* < 0.001 for all).

**Table 1:** Characteristics between cases with hepatocellular carcinoma and controls.

	Cases *N* = 4901		Controls *N* = 19604		
Variable	*n*	(%)	*n*	(%)	*P*-value^∗^
Sex					0.99
Female	1351	(27.6)	5404	(27.6)	
Male	3550	(72.4)	14200	(72.4)	
Age group (years)					0.99
20–39	828	(16.9)	3312	(16.9)	
40–64	1820	(37.1)	7280	(37.1)	
65–84	2253	(46.0)	9012	(46.0)	
Age (years), mean ± standard deviation^†^	62.5 ± 12.3		62.3 ± 12.4		0.44
Ever use of selective serotonin reuptake inhibitors	371	(7.57)	1187	(6.05)	0.001
Other medications					
Ever use of metformin	1173	(23.9)	2827	(14.4)	<0.001
Ever use of statin	581	(11.9)	3646	(18.6)	<0.001
Comorbidities before index date					
Alcohol-related disease	799	(16.3)	998	(5.09)	<0.001
Cardiovascular disease	1653	(33.7)	6647	(33.9)	0.81
Chronic kidney disease	511	(10.4)	1295	(6.61)	<0.001
Chronic liver disease	4110	(83.9)	3029	(15.5)	<0.001
Chronic obstructive pulmonary disease	911	(18.6)	3233	(16.5)	<0.001
Diabetes mellitus	990	(20.2)	1898	(9.68)	<0.001
Hyperlipidemia	1137	(23.2)	5905	(30.1)	<0.001
Hypertension	2454	(50.1)	9316	(47.5)	<0.001

*Data are presented as the number of subjects in each group with percentages given in parentheses. ^∗^Chi-square test, and ^†^*t*-test comparing cases with hepatocellular carcinoma and controls.*

### Association between Selective Serotonin Reuptake Inhibitors Use and Hepatocellular Carcinoma

In **Table 2**, all variables were initially included in the univariable logistic regression model. Only those significantly associated with hepatocellular carcinoma in the univariable logistic regression model could be further included in the multivariable logistic regression model. Therefore, only metformin use, statin use, alcohol-related disease, chronic kidney disease, chronic liver disease, chronic obstructive pulmonary disease, diabetes mellitus, hyperlipidemia, and hypertension could be further included for adjustment. After adjustment for co-variables, the multivariable logistic regression model showed that the adjusted OR of hepatocellular carcinoma was 0.92 for subjects with ever use of selective serotonin reuptake inhibitors (95% CI 0.79, 1.08), comparing with never use. The statistical power of our study to detect a clinically important difference is small (statistical power = 0.24).

**Table 2:** Odds ratio and 95% confidence interval of hepatocellular carcinoma associated with selective serotonin reuptake inhibitors use by logistical regression model.

	Crude		Adjusted^†^	
Variable	OR	(95% CI)	OR	(95% CI)
Ever use of selective serotonin reuptake inhibitors (never use as a reference)	1.27	(1.13, 1.43)	0.92	(0.79, 1.08)

^†^*Initially, all variables were included in the univariable logistic regression model. Only those significantly associated with hepatocellular carcinoma in the univariable logistic regression model could be further included in the multivariable logistic regression model. Therefore, only metformin use, statin use, alcohol-related disease, chronic kidney disease, chronic liver disease, chronic obstructive pulmonary disease, diabetes mellitus, hyperlipidemia, and hypertension could be further included for adjustment.*

### Sub-analysis of Association between Selective Serotonin Reuptake Inhibitors Use and Hepatocellular Carcinoma among High Risk Subjects

In **Table 3**, among subjects with any one of comorbidities including alcohol-related disease, chronic liver disease, and diabetes mellitus, the adjusted OR of hepatocellular carcinoma was 0.89 for subjects with ever use of selective serotonin reuptake inhibitors (95% CI 0.75, 1.06), comparing with never use.

**Table 3:** Association between selective serotonin reuptake inhibitors use and hepatocellular carcinoma among high risk subjects.

Selective serotonin reuptake inhibitors	Any comorbidity^∗^	Case number/control number	Adjusted OR^†^	(95% CI)
Never use	Yes	3922/4479	1.00	(Reference)
Ever use	Yes	339/490	0.89	(0.75, 1.06)

^∗^*Comorbidities including alcohol-related disease, chronic liver disease, and diabetes mellitus. ^†^Adjustment for metformin use, statin use, chronic kidney disease, chronic obstructive pulmonary disease, hyperlipidemia, and hypertension*.

## Discussion

In Taiwan, hepatitis B, hepatitis C, heavy alcohol consumption, and diabetes mellitus are associated with increased risk of hepatocellular carcinoma (Wang et al., 2003; Chen, 2007; Lai et al., 2012). If a person in Taiwan does not have these comorbid conditions, the likelihood of developing hepatocellular carcinoma is low. To the contrary, if a person in Taiwan has one of these comorbid conditions, the likelihood of developing hepatocellular carcinoma is increased. Thus, any study which hopes to explore the drug effect on chemoprevention of hepatocellular carcinoma in Taiwan should focus on persons with any one of these comorbid conditions, not only focusing on the generalized population who does not have these comorbid conditions. Based on this concept, in this study we found that among subjects with any one of these comorbid conditions including alcohol-related disease, chronic liver disease, and diabetes mellitus, selective serotonin reuptake inhibitors use was not significantly associated with of hepatocellular carcinoma (adjusted OR 0.89, 95% CI 0.75, 1.06, **Table 3**). This finding is compatible with a cohort study showing that selective serotonin reuptake inhibitors use was not significantly associated with increased incidence of hepatocellular carcinoma (Haukka et al., 2010). Our finding is not compatible with Chen’s study showing that selective serotonin reuptake inhibitors use was significantly associated with decreased odds for hepatocellular carcinoma (Chen et al., 2017). In addition, our finding was not compatible with an *in vitro* study showing selective serotonin reuptake inhibitors having anti-tumor effects on human hepatocellular carcinoma (Kuwahara et al., 2015). As well-known, only when the result of a study reaches statistically significant, can we need to estimate the statistical power. If the result of a study does not reach statistically significant, the statistical power is usually low. Although the statistical power of our study to detect a clinically important difference is small (statistical power = 0.24), it seems to be accepted. Therefore, inconsistent results exit between the *in vitro* study and the epidemiologic studies. It indicates a future research direction to clarify this issue.

### Strengths and Limitations

There are some strengths in this study. Our study focuses on patients with any one of the comorbid conditions associated with hepatocellular carcinoma. The results are well-structured and explained. The readers are more convinced to the results.

There are some limitations in this study. First, due to the limitation of case-control study, a causal-effect relationship cannot be established. Second, due to the inherent limitation of the database used, the status of alcohol consumption is not recorded. Thus, we used alcohol-related disease for analysis. This point was mentioned in previous studies (Lai et al., 2017c; Lin H.-F. et al., 2017). Third, due to the inherent limitation of the database used, we could not determine whether selective serotonin reuptake inhibitors were used as a mono-therapy for the treatment of depressive disorder or anxiety disorder.

## Conclusion

The findings indicate that among persons with any one of the comorbid conditions associated with hepatocellular carcinoma, no significant association can be detected between selective serotonin reuptake inhibitors use and hepatocellular carcinoma.

## Ethical Considerations

Insurance reimbursement claims data used in this study were available for public access. Patient identification numbers had been scrambled to ensure confidentiality. Patient informed consent was not required.

## Author Contributions

S-WL and K-FL contributed to the conception of the article, initiated the draft of the article, revised the article, and contributed equally to the article. C-LL conducted the data analysis and reviewed the article. H-FL participated in the data interpretation and revised the article.

## Conflict of Interest Statement

The authors declare that the research was conducted in the absence of any commercial or financial relationships that could be construed as a potential conflict of interest.
